# Production of biohydrogen by recombinant expression of [NiFe]-hydrogenase 1 in *Escherichia coli*

**DOI:** 10.1186/1475-2859-9-54

**Published:** 2010-07-07

**Authors:** Jaoon YH Kim, Byung Hoon Jo, Hyung Joon Cha

**Affiliations:** 1 Department of Chemical Engineering, Pohang University of Science and Technology, Pohang 790-784, Korea; 2 School of Interdisciplinary Bioscience and Bioengineering, Pohang University of Science and Technology, Pohang 790-784, Korea

## Abstract

**Background:**

Hydrogenases catalyze reversible reaction between hydrogen (H_2_) and proton. Inactivation of hydrogenase by exposure to oxygen is a critical limitation in biohydrogen production since strict anaerobic conditions are required. While [FeFe]-hydrogenases are irreversibly inactivated by oxygen, it was known that [NiFe]-hydrogenases are generally more tolerant to oxygen. The physiological function of [NiFe]-hydrogenase 1 is still ambiguous. We herein investigated the H_2 _production potential of [NiFe]-hydrogenase 1 of *Escherichia coli in vivo *and *in vitro*. The *hya*A and *hya*B genes corresponding to the small and large subunits of [NiFe]-hydrogenase 1 core enzyme, respectively, were expressed in BL21, an *E. coli *strain without H_2 _producing ability.

**Results:**

Recombinant BL21 expressing [NiFe]-hydrogenase 1 actively produced H_2 _(12.5 mL H_2_/(h·L) in 400 mL glucose minimal medium under micro-aerobic condition, whereas the wild type BL21 did not produce H_2 _even when formate was added as substrate for formate hydrogenlyase (FHL) pathway. The majority of recombinant protein was produced as an insoluble form, with translocation of a small fraction to the membrane. However, the membrane fraction displayed high activity (~65% of total cell fraction), based on unit protein mass. Supplement of nickel and iron to media showed these metals contribute essentially to the function of [NiFe]-hydrogenase 1 as components of catalytic site. In addition, purified *E. coli *[NiFe]-hydrogenase 1 using his_6_-tag displayed oxygen-tolerant activity of ~12 nmol H_2_/(min·mg protein) under a normal aeration environment, compared to [FeFe]-hydrogenase, which remains inactive under this condition.

**Conclusions:**

This is the first report on physiological function of *E. coli *[NiFe]-hydrogenase 1 for H_2 _production. We found that [NiFe]-hydrogenase 1 has H_2 _production ability even under the existence of oxygen. This oxygen-tolerant property is a significant advantage because it is not necessary to protect the H_2 _production process from oxygen. Therefore, we propose that [NiFe]-hydrogenase can be successfully applied as an efficient biohydrogen production tool under micro-aerobic conditions.

## Background

Hydrogenases have been identified in many archaea and bacteria since the initial discovery of the enzyme in *Escherichia coli *[1]. These proteins play important roles in energy metabolism by catalyzing the reversible reaction between hydrogen (H_2_) and proton. Hydrogenases are divided into three classes based on metal content, specifically, [FeFe]-hydrogenases (previously known as [Fe]-hydrogenase), [NiFe]-hydrogenases, and metal-free hydrogenases (now known as [Fe]-hydrogenase) [2]. Among these classes, [FeFe]- and [NiFe]-hydrogenases are the major enzymes; most of hydrogenases found in microorganisms are belong to one of these two enzymes except metal-free hydrogenases that were discovered in some methanogens. The mostly monomeric [FeFe]-hydrogenases are more involved in H_2 _evolution, and display high sensitivity to oxygen (O_2_) and carbon monoxide (CO) (irreversibly inactivated after exposure to O_2_) [3,4]. [NiFe]-hydrogenases, composed of two subunits, are involved in H_2 _oxidation, but also catalyze reversible reactions [5,6]. These enzymes are less active than [FeFe]-hydrogenases by 10~10^2 ^fold, but much more tolerant to O_2 _or CO in a reversible manner [4]. This property is a significant advantage for the biotechnological application of hydrogenase in biohydrogen production, since it is not necessary to protect the production process from O_2_.

Several types of [NiFe]-hydrogenases have been isolated and characterized using genetic and biochemical methods [2,7,8] following the discovery of the enzyme in *Methanobacterium thermoautotrophicum *[9]. In *E. coli*, four types of [NiFe]-hydrogenases have been isolated, and their roles extensively analyzed [10]. Hydrogenase 3 (encoded by *hyc *operon, group 4 [2]) participates in H_2 _production as part of the formate hydrogenlyase (FHL) complex. The FHL complex couples formate oxidation by formate dehydrogenase H (FDH-H) with proton reduction by hydrogenase 3. FDH and hydrogenase 3 are expressed only under strictly anaerobic conditions, and have a highly oxygen-labile nature [2,11]. Accordingly, their application in biohydrogen production using formate as a substrate in anaerobic *E. coli *fermentation has been extensively studied [12]. Hydrogenases 1 and 2 (encoded by *hya *and *hyb *operons, respectively, group 1 [2]) in *E. coli *were isolated by several groups during the mid 1980s [11,13-16]. Results from genetic and physiological studies indicate that hydrogenase 2 is responsible for uptake of H_2 _as an electron donor during anaerobic respiration, with fumarate serving as an electron acceptor or glycerol. This allows cells to obtain energy from H_2 _oxidation [11,13].

The physiological role of hydrogenase 1 is still a matter of dispute. It was reported that active hydrogenase 1 and 2 are produced in *E. coli *during late exponential to early stationary phases when H_2 _concentration would be saturated and hydrogenase 1 could have a role for H_2 _oxidation [14]. Hydrogenase 1 was suggested to participate in recycling of H_2 _produced by hydrogenase 3 [16]. It was also concluded that hydrogenase 1 is not a part of H_2_-evolving FHL system and its role is unclear even though formate is required for expression of hydrogenase 1 [16]. A number of studies have shown that the H_2_-producing ability of a hydrogenase 3 knockout mutant is weak and negligible, leading to the conclusion that hydrogenase 1 only performs H_2 _uptake [17]. In contrast to this conclusion, mutation of hydrogenase 1 did not improve or reduce H_2 _production [17,18] whereas deletion of hydrogenase 2 wholly contributed to increased H_2 _production [13,18]. Hydrogenase 1 and 2 are also immunologically distinct to each other [16]. Thus, it is inaccurate to assume that hydrogenase 1 only exhibits H_2 _uptake activity, similar to hydrogenase 2. In general, the physiological function of hydrogenase 1 is ambiguous [14,18-22].

Hydrogenases, including *E. coli *hydrogenase 1, are uniquely grouped (group 1) based on conserved sequences in their small and large subunits [2]. Using this property, we previously obtained genes for group 1 [NiFe]-hydrogenase core enzyme [23]. Expression of *E. coli *hydrogenase 1 is pH-dependent [19,24], and the enzyme displays a high ratio of H_2 _evolution/H_2 _oxidation for methyl viologen (MV) as substrate under acidic conditions [5,14], similar to another hydrogenase belonging to this group [6]. This finding implies that its reversible reaction is dependent on cellular physiology and possible action as a H_2_-producing enzyme, dependent on the conditions. The enzyme is stable over a wide range of pH values [13,16] and more tolerant to O_2, _compared to hydrogenase 2, FHL, and [FeFe]-hydrogenase [4,14,25-27].

In the present work, we investigated H_2 _production ability of homologously expressed *E. coli *[NiFe]-hydrogenase 1 as a model of group 1 hydrogenase at *in vivo *and *in vitro *status. Through the study, we also try to reveal the basic property of recombinant [NiFe]-hydrogenase 1 related to metabolism, metal effect, and oxygen-tolerance with focusing on H_2 _production.

## Results

### H_2 _production in *E. coli *BL21 by homologous expression of *E. coli *hydrogenase 1

**Figure 1:**
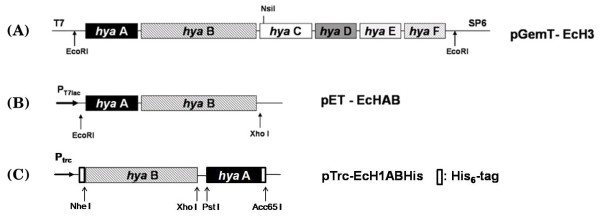
**Scheme for construction of the hydrogenase 1 expression vector**. Full-length *hya *operon was cloned into pGEM-T to generate (A) pGemT-EcH3, and two subunit genes (*hya*A &*hya*B) comprising the core enzyme cloned into pET-21b and pTrcHisC expression vectors to generate (B) pET-EcHAB for the analysis of hydrogen production in recombinant BL21 and (C) pTrc-EcH1ABHis for the analysis of protein expression with His_6_-tag, respectively. His_6_-tag was fused to each subunit gene in pTrc-EcH1ABHis for facile purification of hydrogenase (Refer to Materials and Methods for scheme of His_6_-tag fusion).

**Figure 2:**
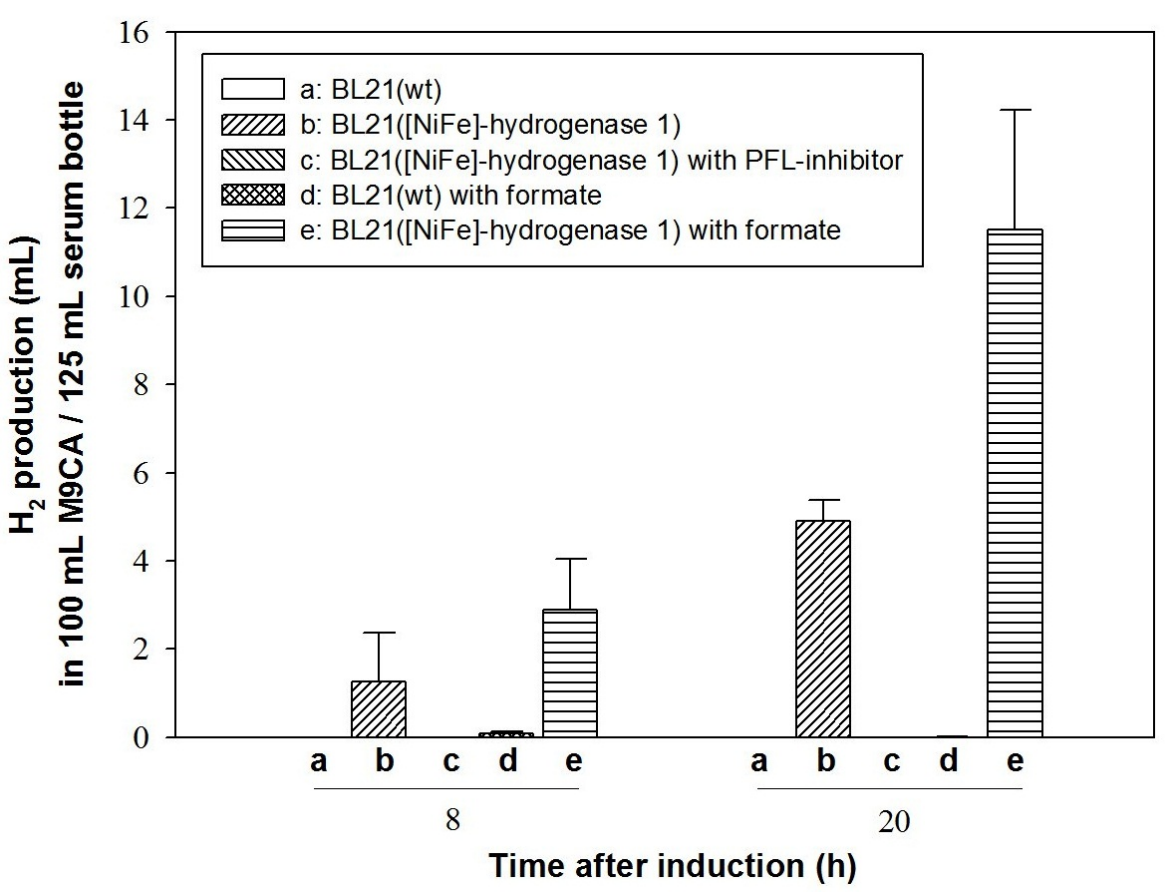
**Analysis of metabolism related to H_2 _production in *E. coli *BL21**. H_2 _production *in vivo *was compared between strains under different conditions. (a) Wild-type BL21, (b) recombinant BL21 expressing hydrogenase 1, (c) recombinant BL21 expressing hydrogenase 1 with addition of 10 mM sodium hypophosphite, (d) wild-type BL21 with addition of 10 mM formate, (e) recombinant BL21 expressing hydrogenase 1 with addition of 10 mM formate. Culture was performed at 37°C and 220 rpm in 125 mL serum bottles containing 100 mL M9CA medium with ampicillin (50 μg/mL) and addition of Ni and Fe at final concentration of 30 μM each, with tight capping for H_2 _measurement in a shaking incubator (seeding and medium preparation were performed in normal aerobic conditions). Serum bottle containing medium was exposed to air for 5 min, and culture performed under micro-aerobic conditions. H_2 _production by hydrogenase 1 was initiated upon induction with 1 mM IPTG, and measured using GC, as described in Materials and methods. Each value and error bar represents the mean of two independent cultures and standard deviation.

Hydrogenase 1 of *E. coli *is transcribed from the *hya *operon composed of six genes (*hya*ABCDEF) [15]. Among these, *hya*A and *hya*B encode the small and large core enzyme subunits, respectively. For expression of hydrogenase 1, the full *hya *operon was initially amplified from the *E. coli *K12 genome, and genes for these core enzyme subunits (*hya*A and *hya*B) cloned under control of the T7*lac *promoter (pET-EcHAB vector; Fig. 1B). *E. coli *BL21 was used as the host strain to overexpress recombinant hydrogenase 1 for investigation of H_2 _production, since this strain does not have ability to generate H_2 _[25,28]. As expected, wild-type BL21 containing the parent pET-21b vector did not produce any H_2 _gas whereas the recombinant strain overexpressing *hya*A and *hya*B actively produced H_2 _in 100 mL culture in a 125 mL serum bottle (bars (a) *vs *(b), Fig. 2). As a comparative study, complete *hya *operon was also overexpressed in *E. coli *BL21. However, we observed no differences from the strain overexpressing *hya*A and *hya*B only (data not shown).

### Effect of endogenous or exogenous Formate on H_2 _production by recombinant *E. coli *hydrogenase 1

Next, we investigated whether H_2 _production by hydrogenase 1 is related to endogenous or exogenous formate and FHL in *E. coli*. H_2 _production in several *Enterobacteriacae*, including *E. coli*, mainly relies on the FHL pathway [29]. Formate is produced *via *pyruvate formate lyase (PFL) from pyruvate under anaerobic conditions. Endogenous formate is decomposed into carbon dioxide (CO_2_) and proton by formate dehydrogenase (FDH-H), and H_2 _generated from the proton by hydrogenase 3 in *E. coli*. We investigated the effect of exogenous formate by adding 10 mM formate to the medium. H_2 _production increased more than 2-fold in BL21 cells with recombinant hydrogenase 1, compared to that in cultures not exposed to formate (bars (e) *vs *(b), Fig. 2). However, wild-type BL21 did not produce H_2_, regardless of addition of 10 mM formate (bars (b) & (d), Fig. 2). This result implies that FHL system of wild type BL21 was impaired. Moreover, since formate is produced from pyruvate, we analyzed the effects of glucose on H_2 _production. The glucose concentration did not affect the H_2 _production to a notable extent. Specifically, we did not observe any noticeable increase in H_2 _production with an increment in glucose concentration from 0.5% to 1.5% (w/v) (data not shown). In addition, we analyzed the effect of endogenous formate on H_2 _production by recombinant hydrogenase 1 using sodium hypophosphite, an inhibitor of PFL. Interestingly, BL21 expressing recombinant hydrogenase 1 did not produce detectable H_2 _following the addition of 10 mM sodium hypophosphite (bar (c), Fig. 2).

### Enzymatic activity and localization of recombinant *E. coli *hydrogenase 1

**Figure 3:**
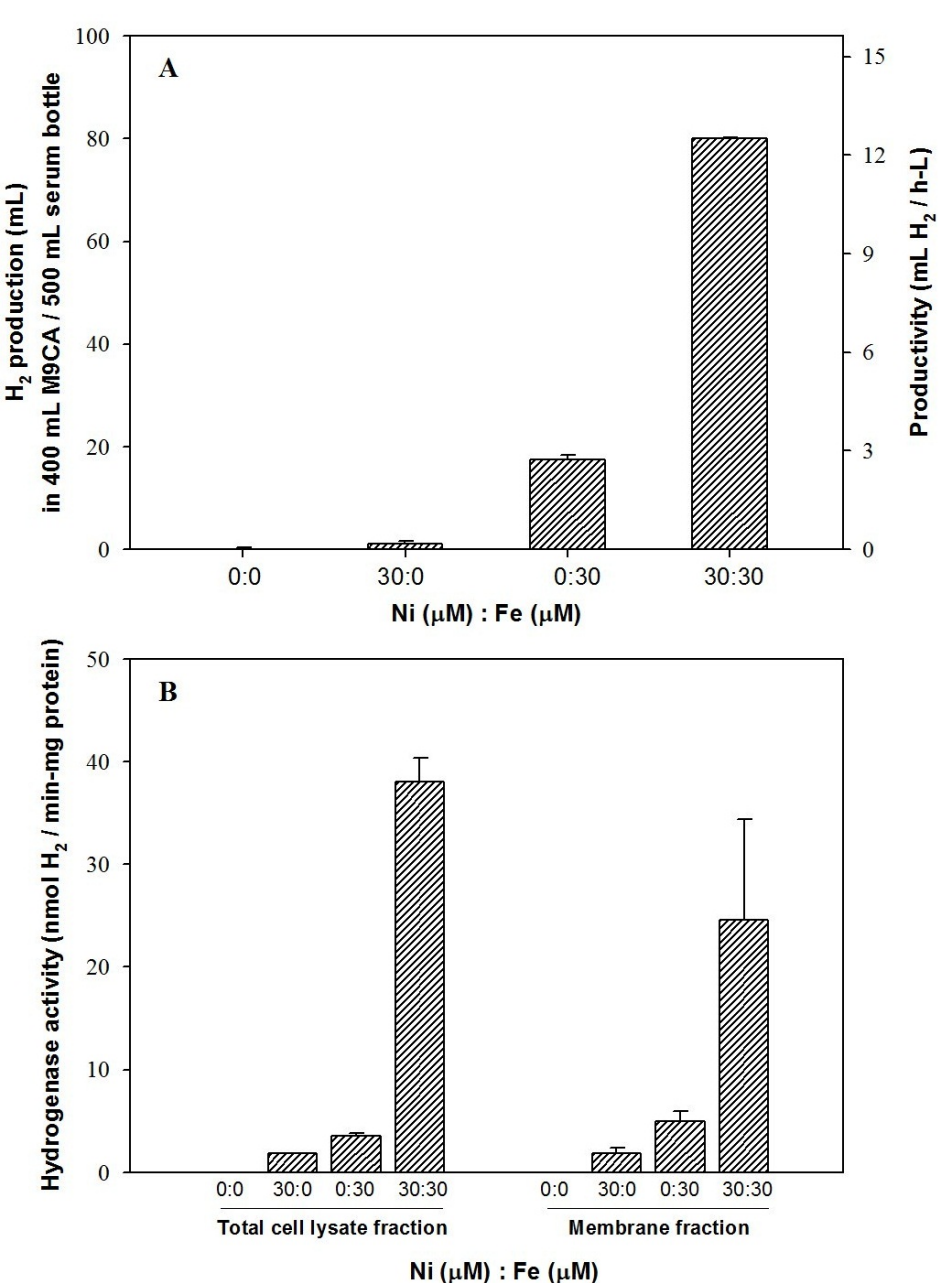
**(A) *In vivo *H_2 _production according to metal content and (B) *in vitro *hydrogenase 1 activity according to metal content and cellular fraction**. Culture was performed in 500 mL serum bottles containing 400 mL M9CA medium. The metal composition was set as four combinations, specifically, (Ni 0 μM, Fe 0 μM), (Ni 30 μM, Fe 0 μM), (Ni 0 μM, Fe 30 μM), (Ni 30 μM, Fe 30 μM). Cell fractionation was performed as described in Materials and methods. Other conditions were similar to those described for Fig. 2. Each value and error bar represents the mean of triplicate samples and standard deviation.

To assess whether expression of recombinant hydrogenase 1 results in a functional enzyme, we assayed enzymatic activity in relation to metal content. We initially investigated metal effects on H_2 _production *in vivo *by altering the ratios of nickel and iron (Fig. 3A). As H_2 _production showed saturated patterns over 20 μM nickel and iron (data not shown), we set each metal concentration as 30 μM in subsequent experiments. Additionally, we used minimal medium, M9CA (M9 media supplemented with casamino acid), to minimize the effects of residual metals on H_2 _production *in vivo *and *in vitro*. Actually, in LB medium, we observed recombinant BL21 produce hydrogen even without addition of Ni and Fe, but there were significant variations according to each culture (data not shown) and it was not so reliable. Upon 400 mL culture in a 500 mL serum bottle in the presence of 30 μM both nickel and iron, recombinant BL21 generated H_2 _most actively (~80 mL) at 16 h post-induction, with productivity of 12.5 mL H_2_/(h·L) (Fig. 3A). Interestingly, H_2 _was generated (~17 mL) with low productivity of 2.7 mL H_2_/(h·L) culture, in the presence of 30 μM iron only. However, upon the addition of 30 μM nickel only, recombinant *E. coli *produced very low amounts of H_2_, and no detectable H_2 _was produced with no addition of both metals. Assessment of the metal content of minimal M9CA medium using atomic absorption spectrometry revealed no Ni and Fe at the detection limit of 0.05 ppm (corresponding to 0.85 μM Ni and 0.89 μM Fe).

To assay the biological activity of recombinant enzyme, we separated the sample into total cell lysate, cell debris, crude extract, cytosolic, and membrane fractions. Comparable to *in vivo *H_2 _production, samples from each culture showed similar hydrogenase activities of *in vitro *H_2 _evolution in both total cell lysate and membrane fractions (Fig. 3B). Total cell lysate and membrane fractions displayed the highest activities (~38 and ~25 nmol H_2_/(min·mg protein), respectively) when supplemented with both nickel and iron. Upon the addition of iron only to the medium, enzyme activities remained at about 9.4% (total cell lysate fraction) and 20% (membrane fraction) of those observed in the presence of both metals. In the case of nickel only, activities of 4.7% and 7.4% were estimated for the total cell lysate and membrane fractions, respectively. No hydrogenase activity was evident in samples prepared without metals. Importantly, the membrane fraction showed relatively high (about 65% with both Ni & Fe) activity per unit mass of total protein compared to the total cell lysate. However, the activity levels of crude extract (substraction of cell debris from the total cell lysate) and cytosolic (substration of crude membrane from crude extract) fractions were significantly lower than those of total cell lysate and membrane fractions (data not shown). Note that we prepared all protein samples for enzyme assay under normal aerobic conditions during harvesting, disruption, and ultracentrifugation. Since the FHL system is easily inactivated under aerobic conditions [11], the H_2 _evolving activity of the cellular extract in the presence of oxygen implies that recombinant *E. coli *hydrogenase 1 displays O_2_-tolerant H_2_-evolving activity.

**Figure 4:**
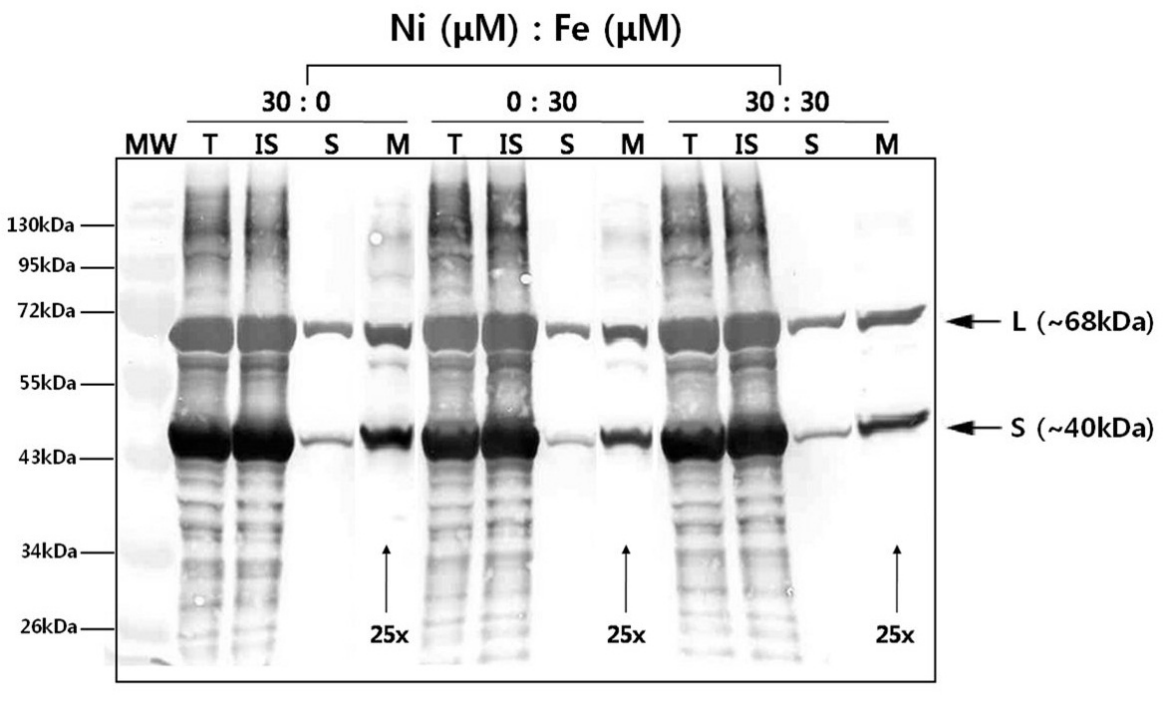
**Western blot analysis of recombinant *E. coli *[NiFe]-hydrogenase 1**. Samples were obtained from different cultures according to their metal compositions, as presented. MW: molecular weight standard marker, T: Total cell lysate fraction, IS: insoluble cell debris fraction, S: soluble crude extract fraction, M: membrane fraction. L with arrow indicates the large subunit of [NiFe]-hydrogenase 1, and S represents the small subunit. 25× indicates 25-fold concentrated membrane fraction, based on the total cell lysate concentration. The protein band was detected with an anti-His_6 _antibody.

[NiFe]-hydrogenase group 1 follows the twin-arginine-translocation (Tat) pathway [2], and localizes to the periplasmic space after maturation and complex formation. Accordingly, we investigated the cellular localization of recombinant hydrogenase 1 of *E. coli *under similar conditions used for analysis of enzyme activity. Total cell lysate, insoluble cell debris, crude extract, and membrane fractions from each culture sample were isolated and analyzed by Western blotting using anti-His_6 _antibody. Large (~68 kDa) and small (~40 kDa) subunits of hydrogenase 1 were overexpressed in the total cell lysate fraction (lane T, Fig. 4). However, the majority of protein was produced as inclusion bodies detected in the insoluble cell debris fraction (lane IS, Fig. 4), and a very small amount of hydrogenase was expressed as the soluble form in the crude extract fraction (lane S, Fig. 4). A significantly low level of hydrogenase 1 was successfully translocated to the membrane and it was detectable after the soluble crude extract fraction was concentrated to 25-fold concentration by ultracentrifugation (lane M, Fig. 4). We observed no significant changes in protein expression upon alteration of the metal content.

In addition, recombinant *E. coli *hydrogenase 1 was purified (data not shown) in the normal aerobic environment using immobilized metal affinity chromatography with the His_6_-tag fused to each subunit (pTrc-Ech1ABHis; Fig. 1C). Hydrogenase evolution activity data showed that purified His_6_-tag fused recombinant [NiFe]-hydrogenase 1 produced ~12 nmole H_2_/(min·mg protein). Notably, enzymatic activity of [FeFe]-hydrogenase was not detected under same aerobic conditions [25].

## Discussion

A previous trial in 1979 demonstrated that purified *E. coli *hydrogenase from aerobic culture could tolerate an aerobic atmosphere with a half-life of 12 h [5]. This hydrogenase was later identified as isoenzyme 1 by immunoblotting after isolation of three types of *E. coli *hydrogenase [14]. A study on *E. coli *hydrogenase 1 expression was performed to determine the roles of proteins comprising the *hya *operon in a *hya *mutant strain [15]. However, due to remaining hydrogenase activities in the *hya *mutant, it was difficult to ascertain the role of hydrogenase 1, and research studies were focused on its H_2 _uptake property. Subsequently, several studies have been performed on hydrogenase 1 in *E. coli *[14,18-22]. Hydrogenase 1 was induced under anaerobic conditions similar to other isoenzymes, but its regulation was distinct from that of hydrogenases 2 and 3 [22]. The enzyme was also induced by formate, similar to FHL, but responded differently to the global regulatory mechanism related to *fnr*. Moreover, hydrogenase 1 had biochemical properties similar to those of hydrogenase 2, but was not immunogenically cross-reactive with hydrogenase 2 [21,22]. The enzyme reversibly catalyzed both dihydrogen evolution and uptake [5,14]. However, a majority of studies concluded that it is not clear to define the physiological role of hydrogenase 1 in *E. coli *or whether it has similar property to hydrogenase 2 or not. Recent reports have shown that H_2 _production was enhanced in deletion mutants of *hya*B and *hyb*C (encoding the large subunit of hydrogenase 2), leading to the proposal that both *E. coli *hydrogenases 1 and 2 have H_2 _uptake activity [17]. However, it remains to be revealed whether H_2 _production is enhanced due to mutation of both hydrogenases or hydrogenase 2 only because another report on this issue suggested that enhancement of H_2 _production is entirely dependent on mutation of hydrogenase 2, but not hydrogenase 1 [18].

In the present work, we investigated the potential of [NiFe]-hydrogenase group 1 as a H_2_-producing enzyme under micro-aerobic conditions using *E. coli *hydrogenase 1 as a model. Although we cloned only the two subunits (small and large subunits) comprising core enzyme of hydrogenase 1, recombinant BL21 successfully produced hydrogen. Large subunit has catalytic site for conversion from hydrogen to proton and electron, and vice versa. Small subunit has [Fe-S] clusters for the transfer of electrons to catalytic site in large subunit. It has been known that accessory proteins are also required for the function of mature [NiFe] hydrogenase 1 [15]. We surmise that there was basal expression of native accessory proteins in the host strain that is required for the assembly and function of *E. coli *hydrogenase 1, and these proteins also functioned on recombinant hydrogenase 1. We initially examined the basic properties of the enzyme in relation to H_2 _metabolism in *E. coli*. Similar to other enterobacteria, *E. coli *utilizes mainly FHL pathway to produce H_2 _from formate, a product of the PFL pathway. Under the addition of formate, H_2 _production increased more than 2-fold in BL21 cells with recombinant hydrogenase 1, compared to the culture without formate (bars (e) *vs *(b), Fig. 2). However, wild-type BL21 did not produce H_2_, regardless of addition of 10 mM formate (bars (a) & (d), Fig. 2) even if formate is known as substrate for FHL complex to produce hydrogen. We also investigated the effect of endogenous formate on H_2 _production by recombinant hydrogenase 1 using sodium hypophosphite, an inhibitor of PFL. Interestingly, BL21 expressing recombinant hydrogenase 1 did not produce detectable H_2 _following the addition of 10 mM sodium hypophosphite (bar (c), Fig. 2). It was also mentioned that lack of H_2 _production ability in BL21 strain might be due to difference in activities of existing hydrogenases [25]. *E. coli *is known to use FHL pathway to produce H_2 _from formate. Nevertheless, we did not notice any meaningful H_2 _production in wild type BL21 even addition of formate while recombinant BL21 with expression of hydrogenase 1 showed highly improved H_2 _production by exogenous formate. Therefore, we surmised this phenomenon strongly support possibility of impaired activity in FHL system. However, further investigation will be required on this point. As mentioned above, it was described that hydrogenase 1 content was also enhanced by exogenous formate even though it was not related to FHL activity and showed different global regulation [22]. Thus, these might be the reasons for improvement of H_2 _production in recombinant BL21 expressing hydrogenase 1. Collectively, these results support that H_2 _production in recombinant BL21, overexpressing hydrogenase 1, depend on function of recombinant hydrogenase 1. Moreover, there have been no evidences or reports that function of hydrogenase 1 could activate FHL complex to produce hydrogen. In addition, formate is produced from pyruvate (the final product of glycolysis), we also checked the effect of glucose on H_2 _production by recombinant hydrogenase 1 for analysis of endogenous formate effect. However, we found that glucose had no noticeable effects on H_2 _production between concentrations of 0.5% and 1.5% (w/v). It seems that there is regulation of internal formate synthesis and its concentration even if more investigation is required. From the results of glucose and sodium hypophosphite, it could be concluded that formate *in vivo *might has a crucial role for production of H_2 _by expression of recombinant hydrogenase 1. We surmised that formate affects H_2 _production by recombinant hydrogenase 1 *via *triggering complex regulation of cellular mechanism and auxiliary proteins that require for hydrogenase function and expression [2]. Besides formate itself, pH change to acidic condition, caused by formate addition and according to culture time, might also influence on H_2 _production by recombinant hydrogenase 1. We consider that more investigation is necessary related to cellular metabolism in future studies.

Since hydrogenase 1 contains nickel and iron at the active site [30], we investigated the effects of these metal ions on expression, *in vivo *H_2 _production, and *in vitro *enzyme activity (Fig. 3). Recombinant cells expressing *E. coli *hydrogenase 1 produced H_2 _most actively upon addition of both Ni and Fe. Cells also produced H_2 _in the sole presence of Fe or Ni in the medium at a lower level than those cultured with both metals, whereas there was no production of H_2 _without addition of both metals. This result could be interpreted that there is critical limit of Ni and Fe concentration required for function of recombinant hydrogenase 1. This result also indicated that addition of Ni and Fe to the minimal media (with no detectable Ni & Fe) have crucial impacts on the function and formation of recombinant hydrogenase 1 because [NiFe]-hydrogenase contains Ni and Fe at the active site in the large subunit and three [Fe-S] clusters with the role of electron transfer in the small subunit. We observed no significant differences in total protein expression upon alteration of metal content (Fig. 4), which might be due to the fact that anti-His_6 _antibody cannot differentiate apoenzyme with holoenzyme and unclear fractionation of recombinant hydrogenase due to overexpression. However, *in vivo *H_2 _production and *in vitro *enzyme activities were significantly affected according to metal addition. *E. coli *hydrogenase 1 exhibits relatively high (~65% compared to total cell lysate) biological activity per unit protein mass in the membrane fraction even if the majority of *E. coli *hydrogenase 1 enzymes were expressed as insoluble forms and membrane translocation efficiency was very low (Fig. 4). Thus, this also implies that correctly translocated enzyme produces high quantity of H_2 _even with very small amount compared to total protein. >From this study, membrane localization of [NiFe]-hydrogenase is very important issue for exhibition of its innate ability. The low translocation efficiency problem should be solved to enhance H_2 _production in the recombinant *E. coli *system.

Importantly, *E. coli *[NiFe]-hydrogenase 1 maintains its biological activity during preparation of the cell extract or purification under a normal aerobic environment as described in Materials and Methods. Our experiments showed that the cell extract maintains *in vitro *H_2 _evolving activity over 10 days at 4°C under aerobic conditions (data not shown). This finding is in agreement with results from previous reports [5], demonstrating that [NiFe]-hydrogenase 1 has oxygen tolerance while [FeFe]-hydrogenase is irreversibly inactivated by oxygen [25]. Accordingly, we propose that [NiFe]-hydrogenase 1 may be successfully used as an efficient biohydrogen production tool under aerobic or micro-aerobic conditions.

## Methods

### Bacterial strains and culture conditions

*E. coli *Top10 (F- *mcrA Δ(mrr-hsdRMS-mcrBC) F80lacZDM15 ΔlacC74 recA1 araD139 Δ(ara-leu)7697 galU galK rpsL *(Str^r^) *endA1 nupG*) (Invitrogen, USA) was used as the host strain for DNA manipulation and cloning of target genes. *E. coli *BL21 (DE3) (Novagen, USA) was used for recombinant expression of the hydrogenase gene. For genetic manipulation, LB medium (Difco, USA) containing the appropriate antibiotics (50 μg/mL ampicillin) was employed. For protein expression, 1 mM (final concentration) isopropyl-β-D-thiogalactopyranoside (IPTG; BioBasic, Canada) was added to each culture medium. For *in vivo *and *in vitro *H_2 _production, cells were grown in M9CA (6 g Na_2_HPO_4_, 3 g KH_2_PO_4_, 1 g NH_4_Cl, 0.5 g NaCl, 5 g casamino acids, and 1 mg/L thiamine supplemented with 2 mM MgSO_4_, 0.1 mM CaCl_2_) medium including 0.5~1.5% (w/v) glucose. All cultures were performed under normal aerobic or micro-aerobic conditions. Cells were grown in a serum bottle (125 or 500 mL; Wheaton, USA) sealed with a rubber stopper and aluminum capping at 37°C in an air shaking incubator (Jeiotech, Korea) at a gyration rate of 200~230 rpm. For micro-aerobic condition, serum bottle containing medium (with empty head space, ~20% of total bottle volume) was exposed to air for 5 min before seeding of cell stock and addition of IPTG. To determine the effects of metal (nickel or iron) on H_2 _production, 0.1 M FeSO_4 _and 1 M NiSO_4 _solutions were prepared, and the appropriate concentrations added to culture medium at the induction point as indicated. The metal concentrations of prepared M9CA before the addition of each metal solution were measured using an atomic absorption spectrometer (with the aid of the Korean Research Institute of Chemical Technology, Korea).

### Vector construction

The *E. coli *K12 MG1655 genome as a template for gene amplification was extracted using the Wizard genomic DNA purification kit (Promega, USA). Primers were designed (upstream; 5'-AAGAGGTATATATTAATGAATAACGAGGAAACATTTTACCAGG-3', downstream; 5'-TTACGTCGGTGCAGCTTCGGCCAGCCACTG-3') based on the sequence from the NCBI database to clone amplified full-length *hya *operon (from *hya*A to *hya*F) into pGEM-T vector (Promega). PCR reaction was performed using Taq polymerase (Takara, Japan) at following condition; 1 cycle of denaturation at 94°C for 5 min (genome)/1 min 40 sec (plasmid template); 30 cycles of denaturation at 94°C for 2 min (genome)/40 sec (plasmid), annealing at 55°C for 40 sec and extention at 72°C for time based on 1 kb/min; 1 cycle of additional extention at 72°C for 5 min. This vector was designated pGem-T/EcH3 (Fig. 1A). After sequencing, the full operon for *hya*A-F was digested from pGem-T/EcH3 with *EcoR*I and cloned into pET 21b (Novagen). A DNA fragment containing *hya*A &*hya*B genes was digested from the constructed vector with *EcoR*I and *Nsi*I (located after the end of the *hya*B open reading frame) and cloned between the *EcoR*I and *Pst*I sites of pSE420 (Invitrogen). Finally, the fragment digested from the constructed vector using *EcoR*I and *Xho*I was cloned into pET-21b, and the construct denoted pET-EcHAB (Fig. 1B). Another expression vector, pTrc-EcH1ABHis, based on pTrcHisC (Invitrogen), was generated for facile purification of recombinant hydrogenase 1 using the His_6 _affinity tag (Fig. 1C). His_6_-tag originally located in pTrcHis vector was fused to N-terminal of large subunit (*hyaB*) and additional His_6_-tag for small subunit was fused to C-terminal of small subunit (*hyaA*) using PCR with primer containing 6×His sequence (CACCATCACCATCACCAC). Direction of His_6_-tag fusion was determined to avoid signal sequence of small subunit at N-terminus and cleavage tail of large subunit at C-terminus.

### Cellular fractionation

Recombinant *E. coli *(400 mL) was harvested by centrifugation at 4°C and 4,000 rpm for 15 min. The cell pellet was resuspended in 50 mM Tris-Cl (pH 6.8) and disrupted with a sonic dismembrator (Fisher scientific, USA) for 10 min at 50% power (5 sec pulse on and 2 sec pulse off). The disrupted cell suspension (total cell lysate fraction) was centrifuged at 4°C and 10,000 *g *for 20 min. The resultant supernatant from previous centrifugation of total cell lysate (crude extract) was centrifuged again at 4°C and 120,000 *g *for 120 min, and the pellet resuspended in 50 mM Tris-Cl (pH 6.8) with weak sonication at 4°C for a few seconds. This fraction was regarded as the crude membrane.

### Affinity purification

After harvesting recombinant *E. coli *grown in M9CA, the cell pellet was resuspended in lysis buffer (50 mM NaH_2_PO_4_, 300 mM NaCl, 10 mM imidazole, pH 8.0) containing 1 mg/mL lysozyme (BioBasic), and incubated for 30 min on ice. After sonic disruption, Ni-NTA agarose slurry (Qiagen, Germany) was added to the cell lysate and mixed by gentle shaking on ice for 60 min. The mixture was loaded on the chromatographic column. After Ni-NTA settled in the column, the resin was washed with buffer (50 mM NaH_2_PO_4_, 300 mM NaCl, 20 mM imidazole, pH 8.0) and bound His_6_-tagged protein to Ni-NTA resin eluted with buffer (50 mM NaH_2_PO_4_, 300 mM NaCl, 250 mM imidazole, pH 8.0). The eluted sample was desalted by several ultrafiltration cycles using a Vivaspin column (MWCO 3,000; Sartorius, Germany) with 50 mM Tris-Cl (pH 6.8) buffer. This desalted and concentrated sample was used later for *in vitro *H_2 _production enzyme activity assay. We performed all of procedures for purification and desalting under normal aerobic condition. In addition, the purified protein sample was analyzed by 15% (w/v) SDS-polyacrylamide gel electrophoresis (PAGE) and 10% (w/v) native-PAGE. For SDS-PAGE, purified protein sample was resuspended in protein sample buffer (0.5 M Tris-HCl (pH6.8), 10% glycerol; 5% sodium dodecyl sulfate (SDS), 5% β-mercaptoethanol, and 0.25% bromophenol blue) and heated to 100°C for 5 min. After centrifugation for 1 min, the samples were loaded onto a 15% (w/v) SDS-polyacrylamide gel for electrophoresis. For solubilization of the membrane fraction containing hydrogenase 1 in native-PAGE analysis, 15 mM n-octyl-β-D-thioglucoside (OTG; BioBasic) was added to the protein sample and incubated at 50°C for 20 min. Electrophoresis was performed in 0.02 M Tris/0.2 M glycine buffer (pH 8.3). The gel was stained with Coomassie Brilliant Blue G-250 (Sigma) for SDS-PAGE and silver nitrate for native-PAGE (Sigma).

### Hydrogenase activity assay

Hydrogenase activity was assayed via *in vitro *H_2 _evolution from MV (Sigma) reduced with sodium dithionite. In a 7 mL sealed vial, a 2.25 mL reaction mixture comprising 50 mM Tris-Cl buffer (pH 6.8), 3 mM MV and prepared enzyme was purged with N_2 _gas for 3 min. The enzymatic reaction was initiated with the addition of 0.25 mL of sodium dithionite (230 mM) at 37°C [31]. H_2 _evolution was measured every 5 min using a gas chromatograph (GC; Younglin Instrument, Korea) equipped with a carboxen-1010 PLOT column (0.53 mm × 30 m; Supelco, USA) and pulsed discharge detector (Valco Instrument, USA). The gas sample (100 μL) was obtained from the reaction vial and injected into GC. Elution was performed using helium as a carrier gas at a flow rate of 10 mL/min, and the temperatures of the injector, detector, and oven set to 130°C, 250°C, and 100°C, respectively. The H_2 _gas level was quantitatively estimated based on the linear relationship between the area of the H_2 _peak and amount of 1% standard H_2 _gas (Supelco).

### *In vivo *hydrogen production

H_2 _gas produced in cell culture was obtained with a gas-tight syringe (Hamilton, USA) from the headspace of a serum bottle (125 or 500 mL) sealed with a rubber stopper and aluminum cap. Usually, 20~100 μL of the gas sample was analyzed using GC. The H_2 _concentration in the gas sample was calculated using a standard curve. The H_2 _amount was determined based on the H_2 _concentration and gas volume of headspace (which expanded when gas was extracted with a syringe).

### Page and Western blot analysis

Samples for each strain with hydrogenase expression were prepared according to cellular fractionation, mixed with protein sample buffer (0.5 M Tris-Cl (pH 6.8), 10% glycerol, 5% sodium dodecyl sulfate (SDS), 5% β-mercaptoethanol, and 0.25% bromophenol blue), and heated to 100°C for 5 min. After centrifugation for 1 min, proteins were separated on a 15% gel. Following SDS-PAGE, gels were transferred to nitrocellulose membranes using a trans-blot cell (Hoefer, USA) and Bjerrum and Schafer-Nielsen transfer buffer (48 mM Tris, 39 mM glycine, and 20% methanol) for 1 h at 50 V and 50 mA. Following transfer, membranes were immunoblotted with monoclonal anti-His_6 _antibody (Applied Biological Materials, Canada; 1:1000 dilution). For primary antibody detection, alkaline phosphatase-conjugated anti-mouse IgG (Sigma) was used as the secondary antibody. After immunoblotting, membranes were washed with TTBS and TBS and developed colorimetrically using nitro blue tetrazolium/5-bromo-4-chloro-3-indolyl phosphate (NBT/BCIP; Sigma).

## Competing interests

The authors declare that they have no competing interests.

## Authors' contributions

JYHK and HJC designed research. JYHK and BHJ performed and analyzed biohydrogen production in recombinant *E. coli*. JYHK and HJC wrote the paper. All authors have read and approved the final version of the manuscript.
